# Changes of physical match performance after the COVID-19 lockdown in professional soccer players according to their playing position

**DOI:** 10.5114/biolsport.2022.114281

**Published:** 2022-04-21

**Authors:** Łukasz Radzimiński, Miguel Lorenzo-Martinez, Marek Konefał, Paweł Chmura, Marcin Andrzejewski, Zbigniew Jastrzębski, Alexis Padrón-Cabo

**Affiliations:** 1 Department of Physiology and Biochemistry, Gdansk University of Physical Education and Sport, Gdansk, Poland; 2 Faculty of Education and Sports Sciences, University of Vigo, Pontevedra, Spain; 3 Department of Biological and Motor Sport Bases, University School of Physical Education, Wrocław, Poland; 4 Department of Team Games, University School of Physical Education, Wrocław, Poland; 5 Department of Recreation, University School of Physical Education, Poznań, Poland; 6 Department of Physical Education and Sport Science, University of A Coruña, 15071, A Coruña, Spain

**Keywords:** Football, Time-motion analysis, Pandemic, High-speed running, Polish Ekstraklasa

## Abstract

The aim of this study was to evaluate the impact of the pandemic lockdown, which included training cessation and home-based training, on physical performance in professional soccer players from the Polish Ekstraklasa according to their playing position. The research was based on 3137 players’ individual match performance observations during the 2019/2020 season divided into before (26 matchdays) and after (11 matchdays) lockdown periods. The data were collected using the computerised multiple-camera optical tracking system TRACAB (ChryronHego VID, New York, NY) with a sampling frequency of 25 Hz. Independently of playing position, a significant (p < 0.001) reduction in season resumption metrics of total distance (-0.9%), jogging (-1.6%), running (-3.4%) and high-speed running (-2.5%), with a contemporaneous increase in walking distance (1.4%), was noted in relation to pre-lockdown performance. A reduction in high-speed running was observed in central defenders (p < 0.05), central midfielders (p < 0.01) and forwards (p < 0.05). No significant decrease in sprinting or maximal running velocity was observed. The COVID-19 lockdown negatively influenced the physical performance in professional soccer players.

## INTRODUCTION

COVID-19 became a worldwide health issue in December 2019. Three months later, on 11 March 2020, the World Health Organization (WHO) declared COVID-19 a pandemic [1]. After this decision, numerous countries implemented restrictions and limitations covering many fields, including sport. Consequently, soccer federations across the world decided to suspend the competition at all levels. Afterwards, the procedures allowing for season resumption were prepared in cooperation with medical committees [2–5]. The typical training sessions for professional soccer players were not allowed as well. In this context, coaches prepared home-based training programmes to maintain the level of players’ physical fitness until the resumption of regular training sessions and official matches. These programmes consisted of such activities as aerobic running, using stationary devices (bike or treadmill), and strength exercises using body weight and small weights.

In European elite soccer leagues, the season is typically divided into three parts: the preseason period, competitive period, and off-season (or transition) period [6]. Also, some leagues use an in-season winter break due to climatic conditions [7]. The off-season period often lasts 4–6 weeks in professional leagues [8], and the length of the in-season winter break varies between 2 and 6 weeks depending on the country [7]. These periods are characterized by a partial reduction of training volume, intensity and different training variation and specificity [6, 9]. It was previously evidenced that both short-term (< 4 weeks) and long-term (> 4 weeks) off-season period types have a detrimental effect on such physical fitness components as body composition, neuromuscular performance (i.e., jump ability, sprint performance, and change of direction), aerobic capacity, and biochemical changes [6, 10]. Therefore, a certain period of appropriate pre-season training is required before resuming the competition.

During the COVID-19 lockdown, a non-planned off-season period occurred in professional soccer. Due to this, the lack of soccer-specific training stimuli was expected to induce a detraining effect, resulting in reduced physical performance [11, 12]. Rampinini et al. [13] reported that home-based training (4–5 aerobic sessions +2 or 3 strength training sessions a week) performed during COVID-19 lockdown had a positive effect on players’ aerobic fitness, although anaerobic power levels were not maintained. Similar results were reported by Grazioli et al. [14] in a sample of Brazilian professional soccer players. Moreover, Parpa and Michaelides [15] confirmed that despite the challenges of the pandemic, properly planned and performed home-based training may significantly improve the level of physical fitness in professional soccer players. In contrast, Albuquerque-Freire et al. [16] found a significant decline in cardio-respiratory capacity expressed by the relative distance covered by players in Yo-Yo intermittent test level 1.

Regarding match locomotion, Santana et al. [17] analysed the match running performance in the German Bundesliga before and after the COVID-19 lockdown. Their results suggest that teams covered a shorter total distance (TD) after the social distancing. However, the number of sprints performed by the players during the game did not change significantly. These findings were confirmed by Radzimiński et al. [18], who compared the physical match performance before and after the lockdown in the German Bundesliga and the Polish Ekstraklasa. They demonstrated that physical match activity in the Polish league was more influenced by the COVID-19 lockdown than the Bundesliga. The shorter period of typical soccer training before competition resumption applied in the Polish Ekstraklasa (only 18 days) was probably one of the reasons for these changes. Similarly, in the Spanish LaLiga soccer-specific team training was allowed only for 14 days before league renewal [19]. As a result, significant decreases in such physical performance variables as total distance, high-speed running distance, sprinting and number of high-intensity actions were noted during the post-lockdown phase of the season.

It is well established within research literature that physical demands differ across playing positions during official soccer matches [20–22]. Thus, the unplanned off-season period may result in changes in ability of position-specific match performance. However, to the authors’ knowledge, none of the previous studies have analysed the changes in match running performance caused by COVID-19 lockdown according to position on the pitch. Therefore, the aim of this study was to evaluate the potential differences in the physical performance of professional soccer players and examine which playing positions were most affected by the COVID-19 lockdown. Based on previous scientific literature [17–19, 23], we hypothesized that professional soccer players would decrease their match running performance after the pandemic lockdown. Furthermore, these physical performance reductions could depend on the playing position.

## MATERIALS AND METHODS

### Experimental approach to the problem

This retrospective, cross-sectional, observational study analysed the effects of the COVID-19 lockdown on the physical match performance of Polish Ekstraklasa players according to playing positions during the 2019–2020 season. In the Polish Ekstraklasa, the pandemic lockdown occurred after completing 26 official matchdays, and 11 matchdays were played after the resumption of the season. In consequence, the season was divided into before and after COVID-19 lockdown periods.

**FIG. 1:**
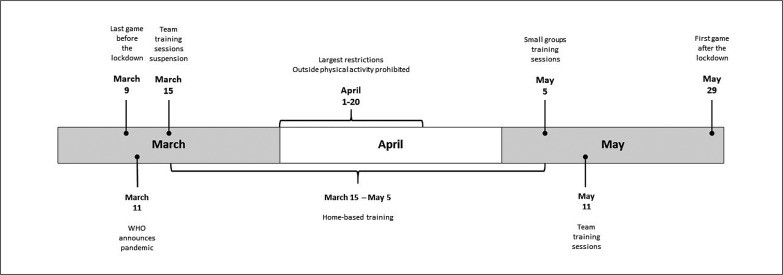
Timeline of the important dates during the COVID-19 lockdown in Polish Ekstraklasa.

The season schedule of the Polish Ekstraklasa established the first games on 19 July and the final matches on 17 May. Due to the pandemic, the season was extended by another nine weeks. On 9 March, the last game before the lockdown was played. From mid March to 5 May, no group training sessions were played. During this period, players performed home-based training involving usually running drills (aerobic running, high-intensity interval sessions), aerobic exercises on the stationary bike and strength training (using body weight and small weights). These training sessions were performed 4–6 times per week. The largest restrictions were enforced by Polish authorities in April, when physical activities were prohibited even outside (in the parks or forest). At the beginning of May, after more than seven weeks, players were allowed to train in small groups consisting of 6–10 players. Such training sessions included numerous technical, soccer-specific drills mixed with conditioning exercises and lasted about 60–75 minutes. Team training sessions restarted on 11 May, which was 18 days before competition resumption. Thus, the pandemic off-season period lasted 81 days [18]. The timeline containing the most important dates during the lockdown in the Polish Ekstraklasa is shown in Figure 1. None of the teams were allowed to play friendly games at this time. All these complications provided numerous difficulties for coaches and sport scientists in preparing players for the final part of the season.

### Participants

The sample was composed of 3137 individual match observations from 209 professional soccer players who participated in official games of the Polish Ekstraklasa in the 2019/2020 season. Data were collected only from outfield players (i.e., goalkeepers were excluded) who completed the entire match. Considering the effect of dismissals in match running performance [24], the matches that involved a player dismissal (i.e., red card) were excluded. Furthermore, only data from players who participated in at least one match before and after the COVID-19 lockdown were included in the final sample. A total of 52 matches were removed due to a dismissal/s from the final sample. Subsequently, the number of observations for the playing position of goalkeeper was 488.

In line with previous studies [16], the playing position was categorized into five positions: central defender (CD), external defender (ED), central midfielder (CM), external midfielder (EM), and forward (F). Playing position was established according to the tactical role on the pitch and the primary area where activity was performed. In order to guarantee player and team confidentiality, all data were anonymized in accordance with the Declaration of Helsinki. This study was conducted in compliance with the local Board of Ethics (agreement number: 12/2021).

### Variables and procedures

Match running performance data were collected using the computerised multiple-camera optical tracking system TRACAB (Chryron-Hego VID, New York, NY) with sampling frequency of 25 Hz. The validity and reliability of this video tracking system have recently been established [25]. In accordance with previous research, match running performance was classified into the following categories: total distance covered (TD), walking (0.7–7.2 km ∙ h^-1^), jogging (7.2–14.4 km ∙ h^-1^), running (14.4–19.8 km ∙ h^-1^), high-speed running (HSR, 19.8–25.1 km ∙ h^-1^), sprinting (> 25.2 km ∙ h^-1^), and top speed reached during the match. The total number of high-speed actions and total number of sprints were defined as the number of high-intensity runs (NHIR). Moreover, the differences between match duration and effective playing time (E_t_) were investigated as well.

The influence of situational variables on match running performance has been well documented [26]. In this regard, the situational variables included in the present study were: match location (home or away), quality of opposition, and match result (win, draw, or lose). In line with previous studies [26], the quality of opposition was defined as the difference in final ranking between teams at the end of the season analysed. All these contextual variables were taken into consideration in creating the model structure.

### Statistical analysis

A linear mixed model was performed to ensure that differences in match running performance between playing positions are attributable to the COVID-19 lockdown. The application of this hierarchical model does not require the same number of measurement points per player before and after the COVID-19 lockdown, unlike a traditional longitudinal statistical model such as analysis of variance (ANOVA) for repeated measures. In addition, the linear mixed model can predict the underlying trends of a particular component of the population (i.e., fixed effects), and model the unexplained variation around the mean trend for that component due to individual team and player differences (i.e., random effects). All analyses were conducted using the statistical software R version 3.6.3 (R Core Team, 2020). The descriptive results for each variable are reported as the means and standard deviations (SD). Linear mixed models were adjusted using the R package “lme4” [27] to analyse the differences between the players’ physical performance before and after the COVID-19 lockdown according to playing position (fixed factors). Additionally, as fixed factors, situational variables such as match location, match status, and quality of opposition were also included in the model. The situational variables were added to the null model and were accepted or rejected based on the model fit determined by the Akaike information criterion (AIC). Player and match identity was modelled as random effects to account for the repeated measurements. Consequently, for each dependent variable (y), the model structure was adjusted:

y=Moment⋅Playing Position+Match Location+Match Status+Ranking Difference+(1|Player ID)+(1|Team ID)

The assumptions of homogeneity and normal distribution of the residuals were established for each model. All models were normally distributed and displayed homogeneous variance. The percentage of change for each variable was also calculated [(After − Before/Before) × 100]. Additionally, effect sizes (ES) were determined using Cohen’s *d*. According to Cohen [28], effect sizes (ES) were classified as trivial (*d* < 0.2), small (0.2 ≤ *d* < 0.5), medium (0.5 ≤ *d* < 0.8) and large (*d* > 0.8). Significance was established at the *p* < 0.05 level.

## RESULTS

The average match duration after the COVID-19 lockdown significantly decreased from 96.9 ± 2.36 to 96.2 ± 2.30 (p = 0.002, ES = 0.30, small) minutes, while E_t_ extended from 55.9 ± 4.74 to 56.3±4.62 (p = 0.38, ES = 0.09 trivial) minutes, which corresponds to 57.7% and 58.5% of match duration respectively.

### Match running performance

Table 1 displays the differences in performance before and after the COVID-19 lockdown in the Polish Ekstraklasa, while Figure 2 depicts the standardized differences (ES) in players’ performance between before and after the unplanned off-season period. Independently of position, players showed significantly less TD (*p* < 0.001), jogging (*p* < 0.001), running (*p* < 0.001), HSR (*p* < 0.001), sprinting (*p* = 0.044), HIR (*p* < 0.001), and NHIR (*p* < 0.001) after the lockdown. In contrast, players increased their distance covered by walking (*p* < 0.001), but no significant effect on players’ top speed was found (*p* = 0.359).

**TABLE 1:** Differences in match-running performance before and after COVID-19 lockdown in Polish Ekstraklasa.

	Independent of playing position			CD			ED		
	Before (n = 2105)	After (n = 1032)	Δ (%)	Before (n = 603)	After (n = 306)	Δ (%)	Before (n = 511)	After (n = 217)	Δ (%)
TD (m)	10698.6 ± 844.7	10604.2 ± 848.6	-0.88*	9930.3 ± 569.0	9834.9 ± 609.1	-0.96*	10695.3 ± 620.7	10611.6 ± 563.4	-0.78*
Walking (m)	3763.3 ± 345.8	3816.2 ± 348.9	1.41*	3852.2 ± 279.8	3899.2 ± 321.1	1.22*	3777.4 ± 374.9	3871.9 ± 327.7	2.50*
Jogging (m)	4336.0 ± 578.2	4265.5 ± 592.6	-1.63*	4071.5 ± 457.2	4036.3 ± 533.4	-0.86*	4280.1 ± 503.2	4106.3 ± 531.4	-4.06*
Running (m)	1767.1 ± 432.2	1707.6 ± 446.8	-3.37*	1418.4 ± 268.0	1344.6 ± 273.4	-5.21*	1681.3 ± 296.6	1664.8 ± 294.1	-0.98*
HSR(m)	669.8 ± 209.6	653.1 ± 212.8	-2.50*	478.8 ± 133.6	451.8 ± 124.1	-5.66^#^	734.3 ± 173.0	741.6 ± 162.2	1.01
Sprinting (m)	162.4 ± 102.3	161.8 ± 104.4	-0.37#	109.3 ± 67.5	103.1 ± 60.7	-5.71	222.3 ± 98.7	227.0 ± 101.6	2.13
HIR (m)	832.3 ± 278.7	814.9 ± 281.7	-2.09*	588.1 ± 177.4	554.8 ± 160.9	-5.66^#^	956.5 ± 236.9	968.6 ± 215.8	1.27
NHIR (n)	56.6 ± 17.4	55.7 ± 17.7	-1.59*	41.2 ± 11.4	39.8 ± 10.5	-3.47	64.6 ± 14.5	65.2 ± 13.3	0.92
Top Speed (km/h)	30.6 ± 1.6	30.6 ± 1.6	-0.01	30.3 ± 1.6	30.2 ± 1.7	-0.49	31.2 ± 1.4	31.1 ± 1.4	-0.18
	**CM**			**EM**			**F**		
	Before (n = 558)	After (n = 268)	Δ (%)	Before (n = 291)	After (n = 154)	Δ (%)	Before (n = 142)	After (n = 87)	Δ (%)
TD (m)	11299.0 ± 726.3	11213.3 ± 707.6	-0.76*	11144.7 ± 675.2	11067.3 ± 695.1	-0.69^#^	10700.3 ± 699.1	10595.4 ± 677.8	-0.98^#^
Walking (m)	3635.9 ± 339.4	3644.5 ± 338.9	0.23*	3729.4 ± 359.4	3782.5 ± 358.7	1.42*	3905.1 ± 316.8	3974.4 ± 295.0	1.77*
Jogging (m)	4694.9 ± 562.2	4682.0 ± 502.1	-0.28^#^	4401.6 ± 599.9	4349.0 ± 596.1	-1.20*	4115.3 ± 560.2	4038.2 ± 470.5	-1.87^#^
Running (m)	2154.8 ± 397.9	2094.3 ± 446.4	-2.81*	1919.8 ± 332.8	1838.4 ± 346.2	-4.24*	1720.1 ± 343.1	1667.9 ± 328.1	-3.04^#^
HSR (m)	693.7 ± 182.8	674.2 ± 183.0	-2.82*	854.7 ± 168.4	846.4 ± 171.0	-0.97^#^	776.3 ± 160.2	732.9 ± 166.0	-5.59^#^
Sprinting (m)	119.7 ± 71.5	118.4 ± 65.2	-1.09	239.3 ± 117.5	251.0 ± 118.4	4.91	183.5 ± 89.2	182.0 ± 100.2	-0.80
HIR (m)	813.4 ± 223.3	792.5 ± 214.0	-2.57*	1094.0 ± 231.7	1097.4 ± 232.7	0.32	959.8 ± 211.4	914.9 ± 224.3	-4.68^#^
NHIR (n)	55.2 ± 13.5	54.1 ± 13.6	-1.94^#^	73.1 ± 14.2	73.3 ± 14.8	0.32	65.8 ± 13.3	62.4 ± 14.9	-5.14*
Top Speed (km/h)	29.9 ± 1.5	30.0 ± 1.5	0.48	31.4 ± 1.5	31.4 ± 1.4	0.19	30.9 ± 1.6	31.0 ± 1.6	0.40

* Significant difference between Before and After (*p* < 0.01);

# Significant difference between Before and After (*p* < 0.05). CD-central defenders, ED-external defenders, CM-central midfielders, EM-external midfielders, F-forwards; TD-total distance, HSR-distance in high-speed running, HIR-distance in HSR and sprinting, NHIR-number of high-intensity runs.

**FIG. 2:**
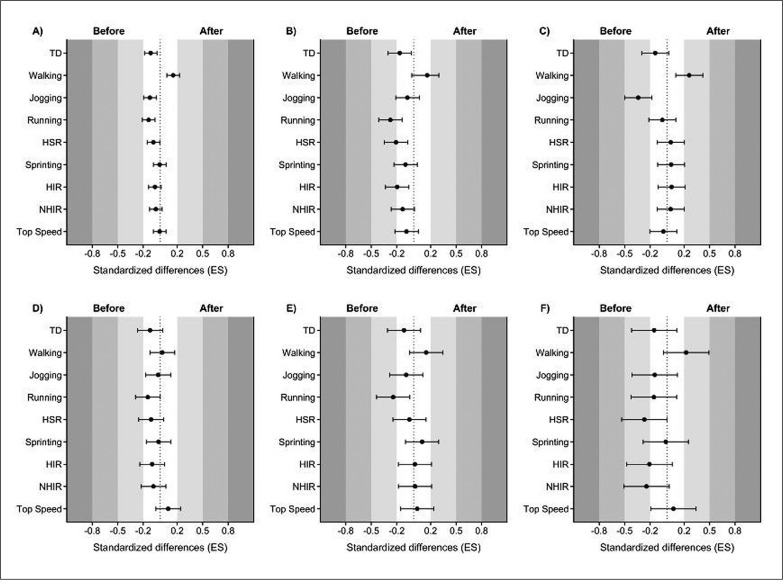
Standardized differences of the physical performance variables according to playing position (A-independent from playing position; B-central defenders; C-external defenders; D-central midfielders; E-external midfielders; F-forwards).

### Positional differences

According to the results displayed in Table 1 and Figure 2, after the COVID-19 lockdown, CD covered significantly shorter TD (*p* = 0.003), jogging (*p* = 0.003), running (*p* < 0.001), HSR (*p* = 0.014), and HIR (*p* = 0.015), but longer distance by walking (*p* < 0.001). With regard to FB, players covered significantly shorter TD (*p* = 0.002), jogging (*p* < 0.001), and running (*p* = 0.008) after the lockdown, while their walking distance increased (*p* < 0.001).

Regarding CM, after the COVID-19 lockdown, players covered significantly shorter TD (*p* < 0.001), jogging (*p* = 0.016), running (*p* < 0.001), HSR (*p* < 0.001), HIR (*p* < 0.001), and NHIR (*p* < 0.001), but longer distance by walking (*p* = 0.003). Regarding EM, players covered significantly shorter TD (*p* = 0.010), jogging (*p* = 0.009), running (*p* < 0.001) and HSR (*p* = 0.040) after the lockdown, while their distance covered by walking increased (*p* < 0.001).

With regard to F, players covered significantly shorter TD (*p* = 0.013), jogging (*p* = 0.026), running (*p* = 0.014), HSR (*p* = 0.021), HIR (*p* = 0.018), and NHIR (*p* = 0.007) after the lockdown, but longer distance by walking (*p* = 0.009).

## DISCUSSION

The current study aimed to establish possible changes in physical match performance in professional soccer players after the COVID-19 lockdown and indicate which playing positions were most affected. In agreement with previous scientific literature [17–19, 23], we hypothesized that professional soccer players would decrease their match running performance after the COVID-19 lockdown. More specifically, this reduction could be position-dependent. The present results indicate trivial-to-small effects on match running performance among Polish Ekstraklasa soccer players. Significant reductions in TD, jogging and running were observed for all playing positions. Moreover, only ED and EM did not show significant decreases in their HSR and sprinting performance. For other positions at least one of these variables was significantly affected.

The influence of the COVID-19 pandemic on the different areas of soccer performance is a scientific topic investigated by several researchers [13, 14]. Brito de Souza et al. [23] reported that during the pre-COVID-19 seasons teams needed 8–10 matches from the beginning of the competition to reach and stabilize their match locomotion. The first games of previous seasons were typically preceded by 12 weeks of the off-season period (including the transition period and 4–6 weeks of the pre-season period). Thus, lower physical match performance after the pandemic lockdown could be partially explained by this effect. Polish Ekstraklasa players resumed their competition after almost 12 weeks of lockdown, while typical team training sessions were allowed 18 days before league resumption. Until this time, players trained in small groups or performed home-based training following the recommendations given by medical committees and scientific evidence [11, 29]. Therefore, negative physical match performance changes after such an in-season break could be expected [23]. Nevertheless, there is no evidence on whether the extension of the team training period would positively affect the match running performance, especially as such a decision could result in higher match frequency after league resumption. Santana et al. [17] reported a significant decrease in TD with a contemporaneous lack of change in the number of sprints performed by German Bundesliga teams after the COVID-19 lockdown. Furthermore, analysis of team performances from the Spanish LaLiga [19] and Polish Ekstraklasa [18] confirmed significant reductions in TD, HSR and NHIR after season resumption. Similarly, the current study showed the significant reduction in TD independently of playing position (-0.9%). The same effect was observed for each position separately. In contrast to the results presented by Santana et al. [17], in Polish Ekstraklasa players match running performance at the highest intensities decreased (HSR: -2.5%; sprinting: -0.4%, and NHIR: -1.6%). However, detailed analysis indicated that this effect was not identical for all positions. Specifically, sprint performance of ED and EM increased by 2.1% and 4.9% respectively. German Bundesliga teams restarted training sessions in small groups 40–44 days before season resumption, while Polish Ekstraklasa teams had only 24 days of training (small-groups and team training) before the first match after the lockdown. Therefore, the longer preparation period in the Bundesliga could help players to avoid the effect of detraining and consequently reductions in physical match performance variables.

The effective playing time was previously identified as one of the factors that should be taken into account when analysing the physical match performance in elite soccer [30]. Thus, eventual reduction in E_t_ could be one of the reasons for lower physical match activity after season resumption. Nevertheless, despite the significant decrease in total match duration, the E_t_ remained stable across pre- and post-lockdown periods (55.9 and 56.3 minutes respectively). Therefore, it can be assumed that E_t_ did not affect the physical match performance during 11 final matches of the analysed season.

Most of the studies investigating physical performance in professional soccer matches usually monitor total distance and distance covered with high intensity, as these variables are considered to be the most important ones [31, 32]. A relatively large number of high-intensity actions requires adequately long recovery periods, during which players try to avoid redundant activity. Walking is an effective movement pattern that allows for fast recovery between high-intensity efforts. As a result, increased frequency of high-intensity actions may require longer distance covered in walking. Significantly longer walking distance covered by the players after the COVID-19 lockdown was observed, while jogging, running and TD decreased significantly. This effect was reported for all the playing positions. Probably maintaining the level of high-intensity performance (e.g. sprinting distance, NHIR) was compensated by a large number of activities with very low intensity. The simultaneous decrease in jogging, running and TD could possibly be caused by a lower level of soccer specific fitness. As a result, players after the lockdown might need more time for recovery between high-intensity actions.

According to Guerrero-Calderón [12], potential muscle disorders generated during the quarantine might affect the ability to perform high-intensity activities and repeated sprints. This assumption was partly confirmed in this study. The present results showed a decline in distance covered in HSR for all playing positions, except ED. Additionally, the effects of the COVID-19 in-season break showed a decrease in distance covered in HIR and NHIR for CM and FW. Meanwhile, the distance covered in sprinting did not change significantly for any of the playing positions. It should be taken into account that these declines in match running performance showed trivial-to-small effects. Although players’ aerobic capacity was not measured in the current study, this fitness component was previously shown to increase due to home-based training strategies during the lockdown [13–15]. In addition, some sports scientists and strength and conditioning coaches emphasized that quarantine caused by pandemic lockdown would lead to a decrease in the ability to generate maximum speed peaks [12]. In the current research, the lack of significant changes in top speed does not seem to confirm these expectations. In a similar context, Requena et al. [8] concluded that a standard off-season period (i.e., 2 weeks of training cessation and 4 weeks of moderate-training load) was effective to maintain sprint performance in top elite professional soccer players. In this regard, a recent systematic review and meta-analysis [10] has suggested that impairments in physical performance could be attenuated using adequate off-season training strategies, which is in line with the results of our study.

Nonetheless, some study limitations should be taken into account. Firstly, the match running performance was analysed using global speed thresholds. It is well established that the lack of speed threshold individualization could generate large differences in quantifying distance covered at high intensity [33]. Furthermore, the presentation of the match running performance does not include values in relation to match duration or E_t_. However, the lack of significant differences in these variables may justify such an approach. Furthermore, some covariates that could potentially influence the results (e.g. matches played in a given week, recovery periods between the games) were not included in the analysis. Additionally, the current study neglects some important and frequent physical actions, such as accelerations and decelerations. Future studies including these variables could be of great interest, since the detraining effects of lockdown on players’ neuromuscular-related qualities were more marked [13, 14]. Moreover, as only the players who completed the entire match were included in the analysis, some players could probably have been substituted due to decreased physical performance [34].. Finally, future research should also consider the effect of such contextual variables as match result, level of opponent and the game location in particular. The lack of spectators on the stadiums after the COVID-19 lockdown disturbed the home advantage phenomenon [35]. Thus, investigating whether this turnover concerned physical match performance as well seems to be an important research topic.

## CONCLUSIONS

The results of the current research indicate that the implemented lockdown significantly reduced match running performance in elite soccer players participating in the Polish Ekstraklasa league. However, the reduction in high-intensity efforts was not equal for all the positions. Moreover, distance covered in sprinting after the lockdown did not change significantly in comparison with the pre-lockdown period. Analysing the influence of such unexpected off-season periods is an important topic and provides practical information for soccer coaches. It is widely known that aerobic capacity is crucial for adequate match performance. Reductions in most of the locomotor variables could suggest an insufficient level of this fitness component after the COVID-19 lockdown. Additionally, shorter distance covered in HSR and lower NHIR could be caused by disturbed recovery ability, which is related to aerobic capacity as well. Therefore, an individualized approach in developing the ability to recover between high-intensity actions should be applied by coaches during home-based training. Moreover, the differences according to playing position underline the necessity of training individualization regarding physical, technical and tactical demands. Fitness coaches should take into account the specific nature of the movement patterns for players from different positions when preparing exercises developing their physical fitness.
